# Tetrodotoxin – Distribution and Accumulation in Aquatic Organisms, and Cases of Human Intoxication

**DOI:** 10.3390/md20080011

**Published:** 2008-05-28

**Authors:** Tamao Noguchi, Osamu Arakawa

**Affiliations:** 1 Tokyo Health Care University, 3-11-3, Setagaya, Tokyo 154-8568, Japan; 2 Faculty of Fisheries, Nagasaki University, 1-14, Bunkyomachi, Nagasaki 852-8521, Japan E-mails: t-noguchi@thcu.ac.jp; arakawa@nagasaki-u.ac.jp

**Keywords:** tetrodotoxin, pufferfish, marine bacteria, newt, gastropod, human intoxication

## Abstract

Many pufferfish of the family Tetraodontidae possess a potent neurotoxin, tetrodotoxin (TTX). In marine pufferfish species, toxicity is generally high in the liver and ovary, whereas in brackish water and freshwater species, toxicity is higher in the skin. In 1964, the toxin of the California newt was identified as TTX as well, and since then TTX has been detected in a variety of other organisms. TTX is produced primarily by marine bacteria, and pufferfish accumulate TTX via the food chain that begins with these bacteria. Consequently, pufferfish become non-toxic when they are fed TTX-free diets in an environment in which the invasion of TTX-bearing organisms is completely shut off. Although some researchers claim that the TTX of amphibians is endogenous, we believe that it also has an exogenous origin, i.e., from organisms consumed as food. TTX-bearing animals are equipped with a high tolerance to TTX, and thus retain or accumulate TTX possibly as a biologic defense substance. There have been many cases of human intoxication due to the ingestion of TTX-bearing pufferfish, mainly in Japan, China, and Taiwan, and several victims have died. Several cases of TTX intoxication due to the ingestion of small gastropods, including some lethal cases, were recently reported in China and Taiwan, revealing a serious public health issue.

## 1. Introduction

**Figure 1:**
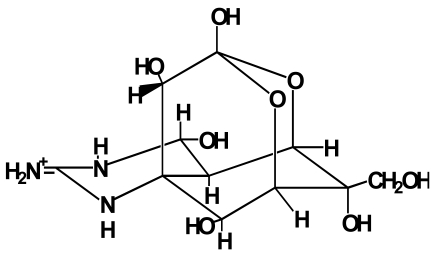
Structure of TTX.

Pufferfish toxin (tetrodotoxin, TTX) is a potent neurotoxin of low molecular weight, which was first isolated in 1950 as a crystalline prism from toxic pufferfish by Yokoo [1]. Its structure (Figure 1) was elucidated by three groups in 1964 [2–4]. TTX inhibits nerve and muscle conduction by selectively blocking sodium channels [5], resulting in respiratory paralysis that causes death. The lethal potency is 5000 to 6000 MU/mg [1 MU (mouse unit) is defined as the amount of toxin required to kill a 20-g male mouse within 30 min after intraperitoneal administration], and the minimum lethal dose (MLD) for humans is estimated to be approximately 10000 MU ( 2 mg) [6]. Various TTX derivatives have so far been separated from pufferfish and/or some other TTX-bearing organisms [7,8].

Many years ago when TTX was believed to be found exclusively in pufferfish, it was controversial whether TTX in the fish was endogenous (produced by the pufferfish itself) or exogenous (taken from the outside and accumulated). Subsequent findings, however, such as the distribution of TTX among many other organisms [9], the TTX intoxication of the trumpet shell following the ingestion of toxic starfish [10], TTX production by marine bacteria [11], and the facts pufferfish become non-toxic when artificially reared with non-toxic diets [12–14] and that such non-toxic pufferfish become toxic when fed TTX-containing diets [15–17], have elucidated that the main mechanism of TTX accumulation in pufferfish is the food chain, consisting of several steps and starting with marine bacteria as a primary source of TTX.

In the present paper, the detailed mechanisms of the distribution and accumulation of TTX are described. We also present some case reports of human intoxication, such as via pufferfish poisoning in Japan, and food poisoning due to the ingestion of small gastropods, which was recently reported in China and Taiwan.

## 2. TTX distribution in aquatic organisms

### 2.1. Distribution of TTX in pufferfish species

The toxicity of the Japanese marine pufferfish was extensively studied by Tani, who reported that 14 of the 21 species examined were toxic [18]. Later, 8 pufferfish species were added to the list of toxic species [19–22], and a total of 22 species are presently listed as TTX-bearing marine pufferfish (Table 1), all belonging to the Tetraodontidae family.

*Lagocephalus wheeleri* and *L. gloveri* of the same family are usually regarded as non-toxic species, although they occasionally show weak toxicity [23]. All species of the Diodontidae and Ostraciontidae families are TTX-free, though the skin of Ostraciontidae contains an ichthyotoxic and hemolytic substance called pahutoxin [24,25]. Food poisoning cases due to ingestion of the liver of Ostraciontid boxfish sometimes occur in Japan, but the causative agent is a toxin with delayed hemolytic activity, possibly palytoxin or a similar substance, and not TTX or pahutoxin [26]. Thai brackish water pufferfish *Tetraodon nigroviridis* and *T. steindachneri*, and freshwater pufferfish *T. fangi*, *T. leiurus*, and *T. suwatii* are also toxic. The toxin of brackish water species was identified as TTX [27,28], but in the freshwater species, saxitoxins (STXs), toxins that belong to the paralytic shellfish poison (PSP) family, were detected as the main toxic principles [29,30]. Freshwater pufferfish, *Colomesus asellus* from Brazil [31] and *T. turgidus* from Cambodia [32], also possess PSP. Recently, some marine pufferfish (*Arothron* spp.) from the Philippines [33], and *Arothron firmamentum* from Japanese waters [34] were also reported to possess STXs as the major toxins. Furthermore, palytoxin-like substance(s), in addition to a small amount of PSP, were detected in a Bangladeshi freshwater pufferfish belonging to the genus *Tetraodon* [35].

The generally large individual and regional variations in pufferfish toxicity [20] are too great to be explained by endogenous factors alone, i.e., differences in physiologic conditions among individual pufferfish, suggesting their exogenous intoxication.

### 2.2. Distribution of TTX in pufferfish bodies

The distribution of TTX in pufferfish bodies appears to be species-specific (Table 1). In marine species, the liver and ovary generally have the highest toxicity, followed by intestines and skin. Muscles and/or testis are non-toxic or weakly toxic, except for in *Lagocephalus lunaris* and *Chelonodon patoca*, and are regarded as edible by the Japanese Ministry of Health, Labour, and Welfare, even in many toxic species. In contrast, in *C. patoca* inhabiting coastal or brackish waters of the Okinawa and Amami Islands [36], and in Thai/Bangladeshi/Cambodian brackish water and freshwater pufferfish, the skin has the highest toxicity [27–29,37].

Our recent immunohistochemical investigations using a monoclonal anti-TTX antibody clarified the intratissue microdistribution of TTX in the skin and ovary of TTX-bearing pufferfish [38–40]. Species-specificity was also recognized in the microdistribution, i.e., TTX was localized in the nucleus, yolk vesicles, and yolk granules of oocytes in the ovary of the marine species *T. vermicularis*, whereas it was detected in the connective tissues and nucleus of some oocytes in *C. patoca* [39]. TTX-bearing glands were observed in the skin of *T. vermicularis*, and TTX-bearing secretory cells (succiform cells) were observed in brackish water species such as *C. patoca*, *T. steindachneri*, and *T. nigroviridis* [38–40].

In the toxic marine pufferfish, the liver generally shows very high toxicity throughout the year except in the spawning season, during which the ovary becomes highly toxic by accumulating TTX transferred from the liver [20]. TTX in the eggs spawned from the ovary could be involved in protecting the eggs from predators. On the other hand, when toxic pufferfish encounter enemies, their bodies swell to two or three times their usual size and TTX is excreted from their skin to repel the enemies [41,42]. These observations suggest that the pufferfish accumulate TTX as a biologic defense agent.

### 2.3. Distribution of TTX in animals other than pufferfish

TTX was long believed to be present only in pufferfish. Since Mosher *et al.* detected TTX in the eggs of the California newt *Taricha torosa* in 1964 [43], however, TTX has been detected in a wide variety of animals, as shown in Table 2 [9].

It is quite unlikely that these TTX-bearers belonging to particular species of different phyla possess a common gene that codes for TTX production. The trumpet shell *Charonia sauliae* accumulates TTX by ingesting toxic starfish [10], supporting the hypothesis that the TTX of pufferfish is not endogenous, but comes via the food chain. The exact origin of TTX in the food chain, however, remains unknown. Because the ecologic environments of TTX-bearing animals seem to have no common factor other than being closely related to an aquatic system, bacteria, omnipresent organisms that commonly inhabit the aquatic system, are implicated as the primary source of TTX.

## 3. Mechanism of TTX accumulation in TTX-bearing organisms

### 3.1. TTX producers

**Figure 2:**
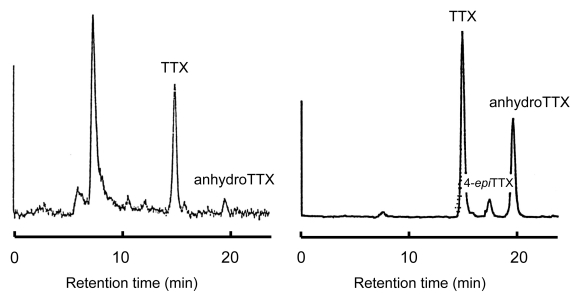
HPLC of TTX fraction from a *Vibrio* strain (left) and of authentic TTX (right) [74].

Based on the above suggestion that bacteria might be the primary source of TTX, in the 1980s we investigated the TTX production of marine bacteria. Intestinal bacteria from several TTX-bearing organisms were isolated, cultured, and examined for lethal potency to mice. *Vibrio alginolyticus* from the toxic starfish *Astropecten polyacanthus* [73,74], one *Vibrio* strain from the xanthid crab *Atergatis floridus* [11,74], and another *Vibrio* strain from the toxic marine pufferfish *Takifugu snyderi* [74] had a paralytic toxicity of 213, 30, and 3 MU per 500 ml of medium, respectively. The toxic principles produced peaks identical to those of authentic TTX and anhydrotetrodotoxin (anhydroTTX) in high performance liquid chromatography with post-column fluorescence derivatization (Figure 2)[74]. In addition, gas chromatography/mass spectrometry analyses revealed that they produce a C_9_-base, the degradation product of TTX, on alkaline hydrolysis [75], leading us to conclude that the isolated bacteria produce TTX. *Shewanella alga* and *Alteromonas tetraodonis* isolated from a red alga *Jania* sp. [76], *Shewanella putrefaciens* from a TTX-bearing marine puffer *Takifugu niphobles* [77], and some other marine bacteria isolated from TTX-bearing organisms [78] also produce TTX.

### 3.2. Mechanism of TTX accumulation in pufferfish

As mentioned above, many years of studies on TTX have revealed that (1) pufferfish toxicity shows remarkable individual and regional variations, (2) TTX is distributed over various organisms, including food animals of pufferfish, (3) the trumpet shell accumulates TTX by ingesting toxic starfish, and (4) marine bacteria primarily produce TTX, suggesting that pufferfish do not synthesize TTX, but accumulate it through the food chain, which starts from marine bacteria. If this is the case, pufferfish should become non-toxic when fed TTX-free diets in an environment in which the invasion of TTX-bearing organisms has been eliminated.

To test this hypothesis, we collected more than 6000 specimens of the marine pufferfish *Takifugu rubripes* that were cultured with non-TTX-containing diets in such environments (net cages on the sea or land aquaria located in eight prefectures of Japan) and examined the toxicity of their livers and some other body parts, including gonads and muscle, using an accepted TTX bioassay [14]. All specimens were non-toxic, i.e., their toxicity was under the detection limit (2–10 MU/g) (Tables 3 and 4). Furthermore, liquid chromatography/mass spectrometry analysis demonstrated that none of the 20 liver specimens from Nagasaki and 5 liver specimens from Yobuko contained detectable amounts of TTX, i.e., their TTX content was below 0.1–0.4 MU/g.

**Figure 3:**
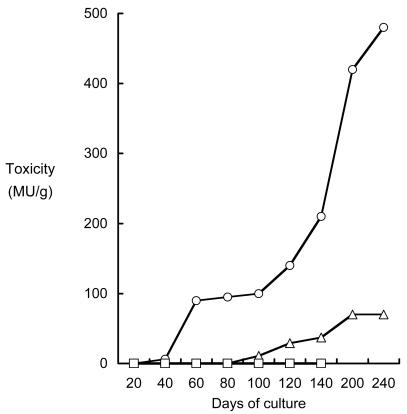
TTX infestation to the non-toxic cultured pufferfish *T. rubripes* and non-toxic three species of fish by feeding TTX-containing livers of wild pufferfish. ▵: Cultured pufferfish; TTX dosage of 0.5 MU/g body mass/day. ○: cultured pufferfish; TTX dosage of 4 MU/g body mass/day. □: non-toxic three species of fish; TTX dosage of 4 MU/g body mass/day.

When non-toxic cultured pufferfish *T. rubripes* were reared on TTX-containing diets, however, they became toxic within 40 days when fed 4 MU TTX/g body mass/day, and in 100 days when fed 0.5 MU TTX/g body mass/day. Thereafter, the toxicity continuously increased up to the end of experimental period (Figure 3) [79]. In contrast, three non-pufferfish species were not intoxicated with TTX, even after culturing for 139 days when fed 4 MU TTX/g body mass/day. These species seem not only to exclude, but also to detoxify, TTX.

In a similar feeding experiment using a PSP [consisting of STX and neosaxitoxin (neoSTX)]-containing diet for 60 days, non-toxic pufferfish were also intoxicated with PSP. Their toxicity scores, however, were only 21% to 53% of those of the fish fed with the same dosage of TTX as controls (unpublished data). Thus, the TTX-bearing marine pufferfish tend to selectively accumulate more TTX than PSP.

**Figure 4:**
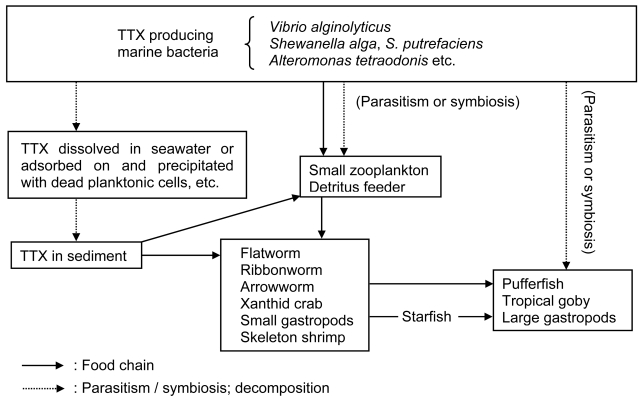
Proposed mechanism of TTX accumulation in marine animals.

These results clearly demonstrate that the TTX of toxic pufferfish is not endogenous, but is derived from the food chain (Figure 4). Another possibility is that the TTX is produced by symbiotic or parasitic bacteria that are directly accumulated inside the puffer body and not obtained via the food chain. The amount of TTX produced by bacteria, however, appears to be too little to account for the TTX accumulation in pufferfish, therefore bioconcentration likely has a large role in the accumulation of TTX in pufferfish. Furthermore, based on the observation that 6000 specimens of pufferfish reared over 1 year in net cages or land aquaria were non-toxic, the TTX produced in the intestines of pufferfish by marine bacteria must be a minor contributor, or rather negligible, to account for the TTX accumulation in pufferfish.

### 3.3. Mechanism of TTX accumulation in amphibians

Some studies of the intoxication mechanism have been performed in amphibians, especially newts. Shimizu and Kobayashi [80] first observed that when various radioactive precursors were administered to two species of newts, *Taricha torosa* and *T. granulosa*, none of the precursors were incorporated into TTX molecules, although the authors did not clearly conclude whether TTX in the newts was endogenous or exogenous. On the other hand, Hanifin *et al.* claim that newts can biosynthesize TTX by themselves, because TTX levels in the dorsal skin of *T. granulosa* significantly increased under artificial rearing with non-toxic diets for 1 year [81], and because the newts released a large amount of TTX from their skin after receiving an electric shock, but the TTX levels in the dorsal skin significantly recovered during the 9 months thereafter [82].

During our studies on the toxicity and toxin profiles of the Japanese newt *Cynops pyrrhogaster* [83,84], we observed that the eggs of the newt retained a certain amount of TTX inherited from their parents, which gradually disappeared in the larval stage. In the juvenile stage in a terrestrial environment, the newt abruptly begins to accumulate TTX again, and becomes highly toxic in the adult stage. When artificially reared with non-toxic diets for 5 years after hatching, however, the newt became non-toxic (< 2 MU/g). The non-toxic newt, when orally administered TTX or 6-*epi*tetrodotoxin (6-*epi*TTX), accumulated approximately 50% of the administered toxins. These observations strongly suggest that the TTX of *C. pyrrhogaster* is exogenous, i.e., the newt begins to accumulate TTX after metamorphosis, with their food organisms as its origin.

Daly *et al.* [85] examined the intoxication mechanism of frogs. They hatched eggs of the Panamanian frog *Atelopus varius* which possesses both bufadienolides (a type of steroid) and TTX in the skin, and artificially reared them for 25 to 45 months. In the skin of the adult frogs, bufadienolides were detected at the same level as in wild specimens, but TTX was not detected, leading them to conclude that bufadienolides are endogenous and the TTX is exogenous, possibly derived from food organisms or environmental factors such as symbiotic bacteria.

### 3.4. Resistance of animals to TTX

Three species each of toxic pufferfish [*T. niphobles*, *T. pardalis*, and *T. rubribes* (cultured specimens)], non-toxic pufferfish (*L. wheeleri*, *L. gloveri*, and *Liosaccus cutaneus*), and non-toxic general fish (*Oplegnathus punctatus*, *O. fasciatus*, and *Girella punctata*) were examined for their resistance to TTX [86]. The MLD of TTX administered intraperitoneally to the three toxic species of pufferfish was 300 to 750 times greater than that of mice (Table 5). Due to this high resistance, the *Takifugu* species can accumulate huge amounts of TTX. Non-toxic pufferfish showed 13 to 15 times stronger resistance against TTX than mice. These pufferfish are generally non-toxic, but can become weakly toxic in some habitats [23]. They can accumulate a limited amount of TTX due to the medium level of resistance to TTX. In contrast, general non-toxic fish had the same low resistance levels as mice, i.e., they cannot accumulate TTX. The goby *Yongeichthys criniger* (formerly *Gobius criniger*) also has high resistance to TTX, and can accumulate TTX [63]. TTX-bearing xanthid crabs [87] and the Japanese newt [84] exhibit even greater resistance to TTX than pufferfish. Based on these findings, it is clear that for TTX-bearing animals to accumulate a certain level of TTX in their body, they must be endowed with high tolerance to TTX.

The mechanism of TTX resistance in pufferfish and rewts has been explained based mainly on the TTX-resistant sodium channels found in the animals, in which the aromatic amino acid commonly located in the p-loop region of domain I in TTX-sensitive sodium channels is replaced by a nonaromatic amino acid, resulting in their extremely low affinity to TTX [88–91]. Garter snakes and clams can also acquire similar sodium channel mutation-based TTX/PSP resistance by interacting with their toxic food organisms, TTX-bearing newts and PSP-producing dinoflagellates, respectively [92,93]. On the other hand, the shore crab *Hemigrapsus sanguineus* possesses TTX-binding proteins in its hemolymph, which confer TTX resistance on this non-toxic crab [94,95]. Pufferfish and newts may be equipped not only with TTX-resistant sodium channels, but also with other mechanisms whereby they mask and/or modulate TTX molecules inside their body.

## 4. Cases of human intoxication by TTX

### 4.1. Poisonings due to pufferfish

#### 4.1.1. Cases in Japan

Many Japanese know that pufferfish, especially their livers (“kimo”), are very toxic. Nevertheless, there are more than a few “kimo” fans who dare to ingest the liver, believing that the toxin can be eliminated by their own special or traditional detoxification methods. Accordingly, food poisonings due mainly to ingestion of the liver were occurring very frequently in Japan [6]. This situation prompted the Japanese Ministry of Health and Welfare (presently the Ministry of Health, Labour, and Welfare) to publish a guideline for edible pufferfish in 1983, with updates in 1993 and 1995 (Table 6), and to prohibit pufferfish liver from being served in restaurants or in the market. Many cases of puffer poisoning, however, continue to occur every year due to the consumption of homemade “kimo” dishes, which are prepared using wild fish that is not purchased commercially. In Japan, over the past 13 years (1995–2007), mortality due to pufferfish poisoning has been 6.4% (Table 7).

##### Case report of pufferfish poisoning [96]

In the early morning of a day in October 1996, a 48-year-old man in Nagasaki, Nagasaki Prefecture, caught a wild marine pufferfish *Takifugu poecilonotus*, and ate more than four slices of slightly cooked “kimo” with some flesh in the evening. Thirty to 60 minutes after ingestion, he began to suffer from numbness in his hands and limbs, followed by cyanosis and respiratory failure during the next 60 minutes. Although immediately hospitalized, he died during the following hour.

In this case, toxicity scores of the leftover liver and flesh were determined to be 715-4260 and less than 5 MU/g, respectively, and TTX was identified as the toxic principle [96]. The victim was estimated to have ingested more than 10000 MU, the amount equivalent to the MLD of TTX in humans, and the cause of his death was concluded to be TTX contained in the liver of wild *T. poecilonotus*.

#### 4.1.2. Cases in Taiwan/China

In Taiwan and China, though people do not eat pufferfish as often as the Japanese, many food poisoning cases due to ingestion of wild pufferfish have also occurred. According to the record of TTX poisoning in Taiwan, there are some cases caused by the mistaken ingestion of muscles of a pufferfish species with toxic muscle, by ingesting puffer roe that had been sold as a fake of dried mullet roe called “karasumi”, or by ingesting a dried dressed fish fillet produced from toxic pufferfish by a food processing company [97–99].

##### Case report of poisoning cases in Taiwan [100]

A food poisoning incident following fish ingestion occurred in Chunghua Prefecture, western Taiwan in January 2000. A total of five victims (4 men, 58–64 years old and 1 woman, 46 years old) were reported. Symptoms included paralysis, coma, nausea, vomiting, ataxia, aphasia, and difficult respiration. Among these victims, two men suffered from more serious symptoms and were treated with intravenous fluids, mechanical ventilation, and intensive treatment in the hospital. They were then discharged uneventfully after 1 week of management.

According to the victims, the causative fish, which had been collected from the coastal area of Chunghua Prefecture, might be the marine pufferfish *Takifugu niphobles*. They retained a small piece (about 11 g) of the cooked fish liver, which, along with eight live specimens of *T. niphobles*, was then assayed for toxicity. The results indicated that the toxicity of the cooked fish liver was 280 MU/g, and all the *T. niphobles* specimens had high toxicity (>850 MU/g) in their liver [100]. The toxin of both samples was identified as TTX. Mitochondrial DNA of the leftover and *T. niphobles* specimens showed the same sequence genotype and the same single restriction site for BsaI, indicating that *T. niphobles* was responsible for the intoxication caused by TTX [101].

#### 4.1.3. Cases in other countries

Ten human fatalities attributable to the consumption of marine pufferfish have been reported in the United States [102], among which four cases occurred in Hawaii during 1908–1925. The *Arothron* sp. was responsible for all the poisonings [103]. In 1986, a further poisoning incident occurred in Hawaii due to consumption of the liver of *Diodon hystrix*, affecting one man [104]. Although he was not admitted to the hospital until 24 hours after exposure, he recovered within 1 week. There is also a case, in which two Dutch sailors died within 17 to 20 minutes after ingesting the liver of a South African marine pufferfish [6].

In Bangladesh, a food poisoning incident due to ingestion of the roe of the marine pufferfish *Takifugu oblongus* occurred in November 1998, affecting eight people and resulting in five deaths [105]. Their symptoms were dyspnea, numbness of the lips, paralysis, and stomachache followed by vomiting, which appeared 2 hours after ingestion. Two victims became unconscious within 5 hours of exposure. On the way to the hospital, two of the eight patients died and the remaining six were admitted. Three of the six patients died in the hospital and three recovered.

Small-sized brackish water and freshwater pufferfish have also occasionally caused food poisoning incidents, including fatal cases in Asian countries such as Thai, Bangladesh, and Cambodia [32,106,107], though the causative toxin is PSP or palytoxin-like toxin in freshwater species.

### 4.2. Poisoning due to small gastropods

In China and Taiwan, food poisoning incidents attributable to the ingestion of small gastropods such as *Niotha clathrata*, *Natica vitellus*, *Oliva miniacea*, *O. hirasei*, *Nassarius glans*, and *Zeuxis samiplicutus*, occur frequently [108–112]. In China, more than 300 people were poisoned, and 16 died during 1977–2001. In June 2001, a poisoning incident due to ingestion of a small gastropod of the family Nassariidae, *Z. samiplicutus* occurred in South Zheijiang, Mainland China. About 30 victims were reported to have symptoms, including paralysis, coma, nausea, vomiting, ataxia, and aphasia. The causative agent was identified as TTX by thin-layer chromatography, electrophoresis, and high performance liquid chromatography [113,114].

It is astonishing that there continues to be many victims, as well as deaths, due to the ingestion of commercially cooked small gastropods in China, which may become a serious public health issue. Moreover, this type of poisoning has spread from the coast of Zheijiang to other areas of the East China Sea and Yellow Sea. Very recently (July 2007) in Nagasaki, Japan, a food poisoning incident occurred due to the same type of small gastropod, *Alectrion glans*. An investigation revealed that a large amount of TTX (600 MU/g) remained in the leftover cooked gastropods, including muscles and digestive glands (unpublished data). Although the TTX accumulation mechanism in the gastropods remains uncertain, we suspect that the gastropods might become toxic by feeding on the toxic viscera of pufferfish that died after spawning.

### 4.3. Poisoning due to other TTX-bearing animals

In December 1979, a 41-year-old man ate a boiled digestive gland (about 60 g) of the trumpet shell *Charonia sauliae*, which he had caught in the coastal waters of Shimizu, Shizuoka Prefecture. Thirty minutes after ingestion, he was hospitalized and received artificial respiration. Although he remained unconscious for 2 days, he recovered fully in 5 days.

The leftover digestive gland had a toxicity score of 17000 MU, and the responsible toxin was identified as TTX [47]. Thus, it was estimated that the victim might have ingested an amount of TTX almost equivalent to the MLD. TTX was also identified as the causative agent in the leftovers from similar TTX poisoning cases in Wakayama Prefecture in 1982 and Miyazaki Prefecture in 1987 [115].

Poisonings due to the ingestion of eggs of the horseshoe crab *Carcinoscorpius rotundicauda* have occasionally been reported in Thailand [116]. The symptoms of the victims were mostly similar to those caused by TTX or PSP. *C. rotundicauda* toxin consists mainly of TTX and anhydroTTX [59,117,118], but sometimes contains STX and neoSTX [119]. Hence, the responsible toxin for horseshoe crab poisonings is considered to be TTX and/or PSP.

## 5. Conclusion

As described above, a large variety of organisms besides pufferfish possess TTX. By knowing the inter- or intra-tissue distribution of TTX, it is possible to estimate the functions of TTX in these organisms. For example, marine pufferfish (Table 1) and flatworms [9,120] retain high toxicity in their eggs, and pufferfish and newts are equipped with TTX-secreting glands or cells in their skin [38–40,121], suggesting that TTX is present as a defensive substance to protect their eggs or themselves from external enemies. In this context, we recently found that the immunity of cultured pufferfish is significantly activated when they are reared with TTX-containing diets [122], though the mechanism remains to be clarified. On the other hand, blue-ringed octopus and ribbon worms possess TTX in the post salivary gland and proboscis, respectively [54,120], indicating that they utilize TTX to capture their prey.

The TTX accumulation mechanism in pufferfish can be explained by bioconcentration via the food chain starting from bacteria. It remains to be elucidated how TTX molecules are absorbed, transferred, and retained/eliminated after entering into the pufferfish body via the toxic food organisms. We recently found that when intramuscularly administered to the non-toxic cultured specimens of TTX-bearing marine pufferfish, TTX is rapidly transferred to the skin and liver through the blood (unpublished data). A similar transfer is also observed when PSP is administered to the non-toxic cultured specimens of PSP-bearing freshwater pufferfish [32]. On the other hand, Nagashima *et al.* [123,124] reported that liver tissue slices of pufferfish accumulate considerable amounts of TTX *in vitro* when incubated with TTX-containing medium. These phenomena were not observed in non-toxic fish, suggesting the presence of a specific mechanism in the pufferfish liver or skin whereby the fish selectively and/or actively accumulate TTX/PSP. TTX-binding proteins exist in the blood plasma of pufferfish [125,126], which may be implicated in the transportation of TTX. In this connection, Lee *et al.* [127] identified novel genes that are possibly related to TTX accumulation in pufferfish.

Not only pufferfish, but various other animals induce TTX intoxication in humans. The symptoms in the patients appear shortly after ingestion of the causative food, and the time to fatality is also very short (average 6 hours). There are currently no antidotes or antitoxins to TTX, and there is no treatment for the seriously TTX-intoxicated patient except for artificial respiration. A monoclonal anti-TTX antibody was recently developed [128], but it can be used only as a reagent for research, and is not expected to have clinical efficacy. There is a possibility that TTX poisoning may increase or diversify further in the future due to changes in the marine environment and the increase in international commerce of fishery products.

TTX has a very important role in neurophysiology, and in other fields of study as a research reagent (a specific sodium channel blocker). In China, it is used as a clinical medicine (an analgesic for terminal cancer patients). In Japan, TTX was previously clinically applied as an analgesic for neuralgia and rheumatism. A thorough understanding of the biochemical properties and mechanisms of TTX accumulation will facilitate the development of technology to use TTX or TTX-bearing organisms effectively as reagents and medicines, or to produce non-toxic cultured pufferfish whose liver can be used as a poison-free food.

## Figures and Tables

**Table 1:** Toxicity of pufferfish

			Maximal toxicity*						
Family	Habitat	Species	Ovary	Testis	Liver	Skin	Intestine	Muscle	Blood
Tetraodontidae	Marine	Japanese pufferfish							
		*Takifugu niphobles*	●	○	●	◎	●	○	—
		*T. poecilonotus*	●	◎	●	◎	◎	○	—
		*T. pardalis*	●	○	●	◎	◎	×	×
		*T. snyderi*	●	×	●	◎	◎	○	—
		*T. porphyreus*	●	×	●	◎	◎	×	—
		*T. chinensis*	●	—	●	—	—	—	—
		*T. obscurus*	●	×	◎	◎	◎	×	—
		*T. exascurus*	●	×	◎	◎	—	×	—
		*T. pseudommus*	●	×	○	○	○	×	—
		*T. chrysops*	◎	×	◎	◎	○	×	×
		*T. vermicularis*	◎	×	◎	◎	○	×	—
		*T. rubripes*	◎	×	◎	×	○	×	×
		*T. xanthopterus*	◎	×	◎	×	○	×	—
		*T. stictonotus*	◎	×	◎	○	×	×	—
		*Tetraodon alboreticulatus*	●	—	○	○	◎	○	—
		*Pleuranacanthus sceleratus*	●	—	○	○	◎	○	—
		Chelonodon patoca**	◎	◎	◎	●	—	◎	—
		*Arothron firmamentum*	◎	×	×	○	×	×	—
		*Canthigaster rivulata*	×	—	○	◎	○	×	—
		*Lagocephalus lunaris*	×	×	×	◎	×	●	—
		*L. inermis*	×	×	◎	×	×	×	—
		*L. wheeleri*	×	×	×	×	×	×	—
		*L. gloveri*	×	×	×	×	×	×	—
		*Sphoeroides pachygaster*	×	×	×	×	×	×	—
	
	Marine	Chinese pufferfish							
		*Takifugu flavidus*	●	◎	●	◎	◎	○	—
	
	Brackish	Thai pufferfish							
		*Tetraodon nigroviridis*	—	—	×	◎	○	○	—
		*T. steindachneri*	—	—	×	◎	×	×	—

Diodontidae	Marine	Japanese pufferfish							
		*Diodon holocanthus*	×	—	×	×	×	×	—
		*Chilomycterus affinis*	×	—	×	×	×	×	—

Ostraciidae	Marine	Japanese pufferfish							
		*Ostracion immaculatum*	×	×	×	×	×	×	—
		*Lactoria diaphana*	×	×	×	×	×	×	—
		*Aracana aculeata*	×	×	×	×	×	×	—

* ×: <10 MU/g tissue; ○: 10–100 MU/g tissue (weakly toxic); ◎: 100–1000 MU/g tissue (moderately toxic); ●: >1000 MU/g tissue (strongly toxic), where 1 MU (mouse unit) is defined as the amount of toxin that kills a male mouse of ddY strain (20 g body weight) in 30 min after intraperitoneal administration. The amount is equivalent to about 0.2 μg of TTX. —: no data available.

** Marine to brackish water species.

**Table 2:** Distribution of TTX in animals other than pufferfish [9].

Animals		Toxic parts	Maximal toxicity*	Ref
Platyhelminthes				
Turbellaria				
Flatworms	*Planocera* spp.	Whole body	●	[44]
Nemertinea				
Ribbonworms	*Lineus fuscoviridis*	Whole body	●	[45]
	*Tubulanus punctatus*	Whole body	◎	[45]
	*Cephalothrix linearis*	Whole body	●	[46]
Mollusca				
Gastropoda	*Charonia sauliae*	Digestive gland	●	[47]
	*Babylonia japonica*	Digestive gland	○	[48]
	*Tutufa lissostoma*	Digestive gland	◎	[49]
	*Zeuxis siquijorensis*	Whole body	●	[50]
	*Niotha clathrata*	Whole body	●	[51]
	*Niotha lineata*	Whole body	●	[52]
	*Cymatium echo*	Digestive gland	○	[53]
	*Pugilina ternotoma*	Digestive gland	○	[53]
Cephalopoda	*Hapalochlaena maculosa*	Posterior salivary gland (adult)	●	[54]
		Whole body (semi-adult)	○	[55]
Annelida:				
Polychaeta	*Pseudopolamilla occelata*	Whole body	○	[56]
Arthropoda:				
Xanthidae crabs	*Atergatis floridus*	Whole body	○	[57]
	*Zosimus aeneus*	Whole body	○	[58]
Horseshoe crab	*Carcinoscorpius rotundicauda*	Egg	○	[59]
Chaetognatha:				
Arrowworms	*Parasagitta* spp.	Head	▵	[60]
	*Flaccisagitta* spp.	Head	▵	[60]
Echinodermata:				
Starfish	*Astropecten* spp.	Whole body	◎	[61,62]
Vertebrata:				
Pisces				
Goby	*Yongeichthys criniger*	Skin, viscera, gonad	◎	[63]
Amphibia				
Newts	*Taricha* spp.	Skin, egg, ovary, muscle, blood	◎	[43]
	*Notophthalmus* spp.	Skin, egg ovary	○	[64,65]
	*Cynopsis* spp.	Skin, egg, ovary, muscle, blood	○	[66]
	*Triturus* spp.	Skin, egg, ovary, muscle, blood	▵	[64,67]
Frogs	*Atelopus* spp.	Skin	●	[68]
	*Colostethus* sp.	Skin	◎	[69]
	*Polypedates* sp.	Skin	◎	[70]
	*Brachycephalus* spp.	Skin, liver	◎	[71,72]

* Maximal toxicity is shown by the same symbols as in Table 1.

▵ derivatives of TTX were detected (toxicity data are unavailable).

**Table 3:** Toxicity of liver of the pufferfish *T. rubripes* cultured in net cages [14]

Place of culture	Year of collection	Age	Number of collection*	Toxicity (MU/g)
Nagasaki**	2001	1–3	494	< 2
	2002	1–3	1021	< 2
	2003	2	240	< 2
Kumamoto	2001	1–2	829	< 2
	2002	2	587	< 2
	2003	Unknown	145	< 2
Kagoshima	1981–83	1–2	47	< 10
	1990–91	1–2	40	< 5
	2002	2	46	< 2
Ehime	2001	2	379	< 2
	2002	2	140	<2
Fukui	1982–83	1–2	25	< 10
Wakayama	1983	1	12	< 10
	2002	2	81	< 2
Shizuoka	2003	2	70	<2
Unknown	2001	2	101	<2

Total			4257	

About 2500 each of fish were reared in a net cage (10 x 10 x 4 m) floating on the sea, whose bottom was more than 10 m apart from the sea bottom. After 1–3 years of culture, the toxicity of *T. rubripes* liver never exceeded 2–10 MU/g (n=4257).

* Specimens were randomly collected from the several numbers of netcages;

** Partly including the specimens of unknown age.

**Table 4:** Toxicity of liver of the pufferfish *T. rubripes* cultured on land

Place of culture	Year of collection	Age	Number of collection*	Toxicity (MU/g)
Yobuko, Saga	2001	2	114	< 2
	2002	2	228	< 2
	2003	2	358	< 2
	2004	2	349	< 2
	2005	2	100	< 2
	2006	2	250	< 2
	2007	2	266	< 4–8
		2	60	< 2–4

			1725	

About 2500 each of fish were reared in an indoor aquarium of 100 ton with the seawater that was pumped up from the sea, filtered through a membrane, and then disinfected by electrolysis. After 2 years of culture, the toxicity of *T. rubripes* liver never exceeded 2–8 MU/g (n=1725).

* Specimens were randomly collected from the 1–3 aquaria in every winter of 2001–2007.

**Table 5:** Resistance of TTX- and non-TTX-bearing organisms.

	Species	MLD (MU/20 g)*	Ref
TTX-bearing organisms			
Xanthid crab	*Atergatis floridus*	1000	[87]
Tropical goby	*Yongeichthys criniger*	> 300	[63]

Japanese newt	*Cynops pyrrhogaster*	> 10000	[84]
Pufferfish			[86]
Toxic	*Takifugu niphobles*	700–750	
	*T. pardalis*	500–550	
	*T. rubripes* (culture)	300–500	
Generally non-toxic or rarely toxic	*Lagocephalus wheeleri*	15–18	
	*L. gloveri*	19–20	
	*Liosaccus cutaneous*	13–15	

Non-toxic	*Ostracion immaculatum*	0.9–1.3	
TTX-free vertebrates			[86]
Teleosts	*Oplegnathus punctatus*	0.8–0.9	
	*O. fasciatus*	0.8–1.8	
	*Girella punctata*	0.3–0.5	

Land mammal			
Mouse	*Mus musculus*	1	

* MLD of TTX (MU/20 g body mass) that killed 100% of the test animals by intraperitoneal injection.

**Table 6:** Edible part of pufferfish in Japan

Family	Species	Muscle	Edible part Skin	Male gonad
Tetraodontidae	“Kusafugu” *Takifugu niphobles*	○	—	—
	“Komonfugu” *T. poecilonotus*	○	—	—
	“Higanfugu” *T. pardalis*	○	—	—
	“Shousaifugu” *T. snyderi*	○	—	○
	“Nashifugu” T. vermicularisa*	○	—	—
	“Mafugu” *T. porphyreus*	○	—	○
	“Mefugu” *T. obscurus*	○	—	○
	“Akamefugu” *T. chrysops*	○	—	○
	“Torafugu” *T. rubripes*	○	○	○
	“Karasu” *T. chinensis*	○	○	○
	“Shimafugu” *T. xanthopterus*	○	○	○
	“Gomafugu” *T. stictonotus*	○	—	○
	“Kanafugu” *Lagocephalus inermis*	○	○	○
	“Shirosabafugu” *L. wheeleri*	○	○	○
	“Kurosabafugu” *L. gloveri*	○	○	○
	“Yoritofugu” *Sphoeroides pachygaster*	○	○	○
	“Sansaifugu” *T. flavidus*	○	—	—
Diodontidae	“Ishigakifugu” *Chilomycterus reticulatus*	○	○	○
	“Harisenbon” *Diodon holocanthus*	○	○	○
	“Hitozuraharisenbon” *D. liturosus*	○	○	○
	“Nezumifugu” *D. hystrix*	○	○	○
Ostracidae	“Hakofugu” *Ostraction immaculatum*	○	—	○

○ Edible; —, non-edible.

* Applicable to muscle of the species caught in Shimabara Bay, Tachibana Bay and the Inland Sea of Kagawa and Okayama, and to male gonad in Shimabara Bay and Tachibana Bay.

**Table 7:** Pufferfish poisoning incidents in Japan.

Year	Number of incidents	Number of patients	Number of deaths	Mortality (%)
1965	106	152	88	57.9
1970	46	73	33	45.2
1975	52	75	30	40.0
1980	46	90	15	16.7
1985	30	41	9	22.0
1990	33	55	1	1.8
1995	30	42	2	4.8
1996	21	34	3	8.8
1997	28	44	6	13.6
1998	27	39	4	10.3
1999	20	34	2	5.9
2000	29	40	0	0.0
2001	31	52	3	5.8
2002	32	49	5	10.2
2003	28	35	3	8.6
2004	43	58	2	3.4
2005	40	49	2	4.1
2006	25	32	1	3.1
2007	24	38	2	5.3
